# Investigation of ionic liquid adsorption and interfacial tension reduction using different crude oils; effects of salts, ionic liquid, and pH

**DOI:** 10.1038/s41598-024-58458-0

**Published:** 2024-05-10

**Authors:** Mojtaba Khosravani, Naser Akhlaghi, Seyednooroldin Hosseini

**Affiliations:** 1 Department of Chemical Engineering, Omidiyeh Branch, Islamic Azad University, Omidiyeh, Iran; 2 Department of Petroleum Engineering, Omidiyeh Branch, Islamic Azad University, Omidiyeh, 63731-93719 Khuzestan Iran; 3 Department of Petroleum Engineering, Omidiyeh Branch, EOR Research Center, Islamic Azad University, Omidiyeh, Iran

**Keywords:** In-situ surfactant, Saponification, Alkaline, Adsorption, Salting-out, pH, Ionic liquid, Chemical engineering, Chemistry

## Abstract

The results revealed the significant effect of NaCl, KCl, CaCl_2_, MgCl_2_, CaSO_4_, MgSO_4_, and Na_2_SO_4_ and pH values of 3.5–11 on the interfacial tension (IFT) reduction using three types of neutral, acidic, and basic crude oils, especially for acidic crude oil (crude oil II) as the pH was changed from 3.5 to 11 (due to saponification process). The findings showed the highest impact of pH on the IFT of crude oil II with a reducing trend, especially for the pH 11 when no salts exist. The results revealed that the salts except MgCl_2_ and CaCl_2_ led to a similar IFT variation trend for the case of distilled water/crude oil II. For the MgCl_2_ and CaCl_2_ solutions, a shifting point for IFT values was inevitable. Besides, the dissolution of 1-dodecyl-3-methyl imidazolium chloride ([C_12_mim][Cl]) with a concentration of 100–1000 ppm eliminates the effect of pH on IFT which leads to a reducing trend for all the examined crude oils with minimum IFT of 0.08 mN/m. Finally, the [C_12_mim][Cl] adsorption (under pH values) for crude oils using only Na_2_SO_4_ was measured and the minimum adsorption of 0.41 mg surfactant/g Rock under the light of saponification process was obtained.

## Introduction

Chemical injection for higher oil production from depleted reservoirs is an applicable method, especially as the oil price increases. The chemical injection is applicable since it can manipulate mechanisms such as IFT reduction, wettability alteration, viscosity reduction, etc. One of the primary methods proposed for higher oil recovery is alkaline injection since alkalis are cost-effective and have the potential to produce in-situ soap and surfactants at different pH conditions by reacting with the acidic contents of the crude oil. In the light of soap formation several phenomena including emulsification^1^, emulsion formation^2^, changing surface wetness from oil-wet to water-wet^3,4^, or water-wet to oil-wet^5^, coalescence^6^, and sweep efficiency are possible. Unfortunately, using alkaline injection or any chemical that is alkali-like and pH manipulator may have an intrinsic drawback due to the possible precipitation into the solutions containing low or high amounts of divalent ions. Due to this precipitation, the high-permeability channels may be blocked and the sweep efficiency reduction will be inevitable^7^. In this way, investigating the synergy of alkalis with salts is one of the essential stages of using these chemicals for tertiary oil recovery purposes with the assistance of IFT reduction, wettability alteration due to soap adsorption, and capillary number (residual oil saturation) manipulation^8,9^.

For example, Hoeiland and his coworkers^10^ investigated the effect of pH on IFT using acidic crude oil. They reported that not only increasing pH reduced the IFT but also it increased the heavy acidic oil recovery^11–13^. Also, the previously performed investigations revealed that the efficiency of alkaline flooding is highly related to the pH value and other system properties along with the crude oil acidic content^14^. Although there are several reports regarding the undeniable synergy between the pH and acidic contents of crude oil, there is no consistent trend regarding this topic, yet^15–17^.

Besides pH and the crude oil type, the investigations showed that the impacts of salts are significant enough to consider them as a crucial parameter for IFT reduction and wettability alteration especially as the surfactants existed in the solution. In this way, different studies have been performed to study the brine and oil composition impacts along with the thermodynamic conditions to find the possible synergy between chemicals and crude oil compositions. For example, Derkani and his colleague^18^ published a comprehensive survey regarding the interfacial mechanisms for low salinity water (LSW) in carbonate reservoirs that revealed the role of fluid/fluid (IFT reduction) and fluid/solid (wettability alteration) interactions through the enhanced oil recovery (EOR) regardless of the rock type^19,20^. Moreover, the effects of salts from chloride and sulfate families that existed in seawater were examined by Dehaghani and Elyaderani^21^ to see if these salts can change the IFT reduction and wettability simultaneously.

The situation would be more complicated as the surfactant effect combines with the pH, salinity, and pH (presence of alkalis) since it is possible to manipulate the IFT and rock surface wetness simultaneously and effectively^22^. In this way, the application of surfactants for higher oil recovery oil from the carbonate or sandstone reservoirs has been examined by number of researchers^23–27^.

The findings of those investigations revealed that for every reservoir condition, the compatible surfactants are selected after a series of investigations based on the reservoir temperature and salinity, surfactant structure, formation type, pH, the permeability of the rock, cost of the surfactant, adsorption of the surfactant on the matrix rock, and finally, the oil recovery^28^. One of the most relevant problems of the surfactant flooding system is the loss of surfactant due to adsorption in the reservoir since it directly affects the economics of surfactant flooding, and thus it is important to analyze the adsorption properties of the surfactant^29–32^.

In other words, although the surfactants are efficient for oil recovery by activating multiple mechanisms, their stability under harsh salinity and temperature conditions, their retention, and adsorption are limiting factors that need to be investigated^33^. In detail, surfactant injection for higher oil production generally suffers a drawback of a significant surfactant loss due to retention in the porous media^28,34^.

Generally, it is possible to consider the surfactant precipitation, adsorption, and phase trapping as surfactant retention. Among these categories, surfactant retention occurs due to precipitation, and phase trapping can be avoided if a proper surfactant is selected although complete surfactant adsorption is impossible^29,35^.

Amit Kumar et al.^36^ examined the zwitterionic surfactant adsorption on both carbonate and sandstone rock surfaces. Their results revealed that the salinity has a direct effect on the adsorption of surfactant for both studied rock types concomitant with a considerable effect on the rock hydrophilicity. Also, Tariq et al.^37^ investigated the viscoelastic and adsorption behavior of ionic liquid (IL)-based surfactants which showed a low adsorption for ILs. But, the point is that the investigations revealed that it is the surface charge of rocks and surfactants that can control the adsorption overall behavior^38,39^.

So, reducing the adsorption of surfactant on the rock surface and measuring the surfactant adsorption to propose the best chemical formulation to reach the ultimate oil recovery is highly essential since the amount of used surfactant covers about 50% of the chemical injection costs^34,40^. Therefore, it is vital to minimize surfactant adsorption as a key factor in achieving and designing a cost-effective surfactant flooding process^41^. Respecting the importance of this concern, a comprehensive review was performed by Belhaj et al.^42^ to describe the effect of different parameters of salinity, surfactant type, crude oil composition, etc. on surfactant adsorption.

In light of these limitations, the researchers proposed several solutions to overcome such limitations such as using a combination of surfactants with other chemicals such as alkali and polymer as the common combinations. As previously mentioned, alkalis are effective for oil recovery if used concomitantly with surfactant since they can strengthen the impact of surfactants for better IFT reduction and reduce the surfactant adsorption as they produce in-situ soap which can act as sacrifice as they adsorbed on the rock surface before the surfactant injection^43,44^. Among the different possible candidates of surfactants for EOR purposes, ILs are one of the desired options since they can tolerate harsh salinity and temperature conditions^45–48^.

In detail, ILs with hydrophilic heads and long alkyl chains that have amphiphilic character are highly surface active and can be considered as the surface-active ionic liquids known as SAIL^49–51^. In this way, several investigations examined the SAILs to find their advantages over the conventional surfactants including (a) higher functionality of SAILs under high salinity and temperature, (b) it is possible to fabricate and tailor any specific-task IL compatible with the operating conditions of reservoirs, (c) SAILs introduce higher viscosity than traditional surfactants probably is applicable to modify the sweep efficiency of the injecting fluid for EOR purposes, and (d) it is possible to reach low and ultra-low IFT values using only one SAIL which means less risk of using and releasing co-surfactant into the environemnet^52–55^.

For example, Zabihi et al.^56^ examined the effect of conventional (sodium dodecylbenzene sulfonate (SDBS)) compared with the IL-based (imidazolium and pyridinium chlorides) surfactants on IFT reduction and tertiary oil recovery. They reported that both of the examined surfactants can tolerate harsh salinity conditions up to 50,000 ppm although using SDBS concentration higher than 1600 led to fast precipitation in the aqueous solution.

Also, not only the effect of ILs on the IFT reduction or wettability alteration is important to be examined, but it is also highly required to examine the adsorption of the IL on the rock surface. The point is that not only the chemicals have a profound impact on the different mechanisms, but the charge of the rock surface is essential during the adsorption phenomenon. In detail, since IL adsorption is mainly the electrostatic attraction between the charged surface of the solid and the charged head group of the IL molecule this is the solid–liquid interface charge dictates the amount of IL adsorption which is a pH and ionic strength-dependent parameter^57^. In light of this fact, examining the effect of salinity, IL type, pH, etc. is highly required to be examined on the IFT reduction, wettability alteration, and IL adsorption for higher oil recovery purposes.

Respecting these facts, the current work is focused on the effects of sulfate salts (CaSO_4_, MgSO_4_, and Na_2_SO_4_ under low salinity conditions of 1500, 5000, and 5000 ppm, respectively) and chloride salts of CaCl_2_, MgCl_2_, KCl, and NaCl and with concentration of 5000 ppm) and their relation to the pH values of 3.5 (acidic condition), 7 (neutral) and 11 (basic) in contact with three different crude oil samples (acidic, neutral and basic) for the first time since there are no reports regarding the synergy between these chemical and operating condition. Moreover, the current work is intentionally aimed not only to investigate those parameters on IFT reduction but also to find the impact of 1-dodecyl 3-methyl imidazolium chloride ([C_12_mim][Cl]) on the IFT of the optimum formulation concomitant with its adsorption patterns under different pH values for the first time using dynamic adsorption approach. The point is that the current study is focused on using salt concentration under low salinity conditions since the low salinity have gain an increasing attention for EOR purposes during the past decades.

## Materials and methods

### Chemicals

Calcium sulfate (CaSO_4_), sodium sulfate (Na_2_SO_4_), magnesium sulfate (MgSO_4_), sodium chloride (NaCl), calcium chloride (CaCl_2_), magnesium chloride (MgCl_2_), potassium chloride (KCl) with purity better than 99.5% were supplied from Merck, USA. All of these salts were dissolved in distilled water with a concentration of 5000 ppm except CaSO_4_ dissolved with a concentration of 2000 ppm (since CaSO4 dissolution for concnetrations higer than 2100 ppm was impossible due to early precipitation) to mimic the low salinity condition for the targeted solutions. Also, the selected IL which is 1-dodecyl-3-methylimidazolium chloride ([C_12_mim][Cl]) was synthesized according to the procedure previously reported^55^.

### Analysis

The sample crude oils from three different oil reservoirs under the management of the Iranian Offshore Oil Company were kindly provided and used as the sample oils (analyzed by a high-resolution Gas Chromatographic Varian 3400 GC coupled with SARA analysis) (see Table 1). Besides, the total acid numbers (TAN) for the sample oils were measured since they can show the crude oil acidity or neutrality. In detail, the acidic crude oil TAN above 0.5 mg KOH/g^58^ which significantly depends on the carboxyl group content mostly exists in the asphaltenes and resins^59,60^. The TAN of the crude oils is usually calculated using the ASTM D 664 method which is based on potentiometric titration. The examinations revealed that the crude oil II samples which are highly acidic with TAN of 1.63 mg KOH/g oil also contained high contents of asphaltene and resin (9.8 wt% and 11.6 wt%, respectively), while the crude oil III sample had a low acid fraction (TAN = 0.19 mg KOH = g oil) with a low content of asphaltene (4.3 wt%) and resin (7.3 wt.%).

**Table 1:** The gas chromatography (GC) analysis for the sample crude oils.

Componenet	wt% in neutral crude oil (Crude oil I)	wt% in acidic crude oil (Crude oil II)	wt% in Basic crude oil (Crude oil III)
N. Paraffins	6.81	10.97	12.2
I. Paraffins	5.10	7.89	8.91
Olefinic	0.15	0.25	0.06
Naphthenes	6.92	8.33	12.3
Aromatics	7.38	9.10	6.70
others	73.64	63.46	59.83

**Figure 1:**
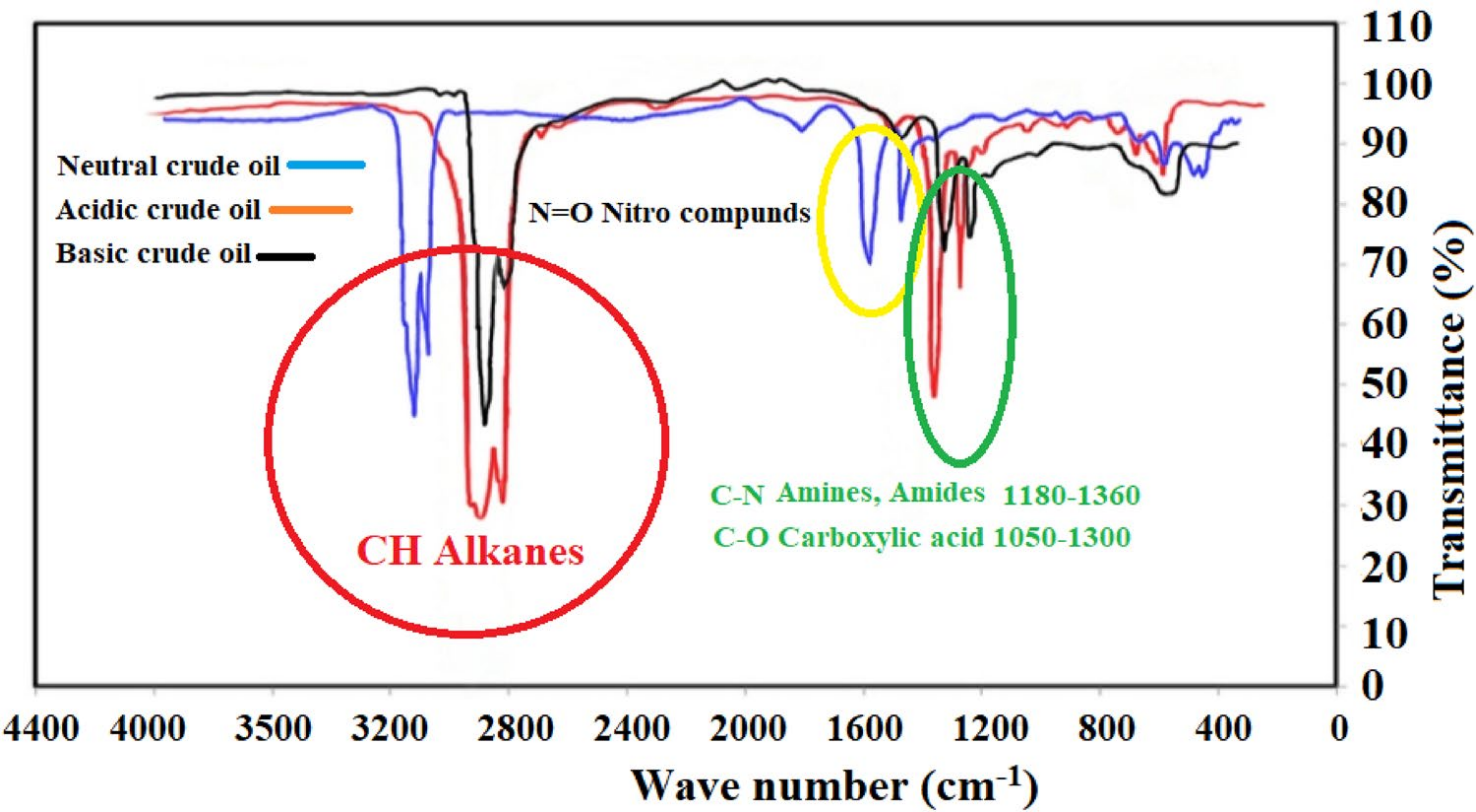
FT-IR spectroscopy for different crude oils.

**Figure 2:**
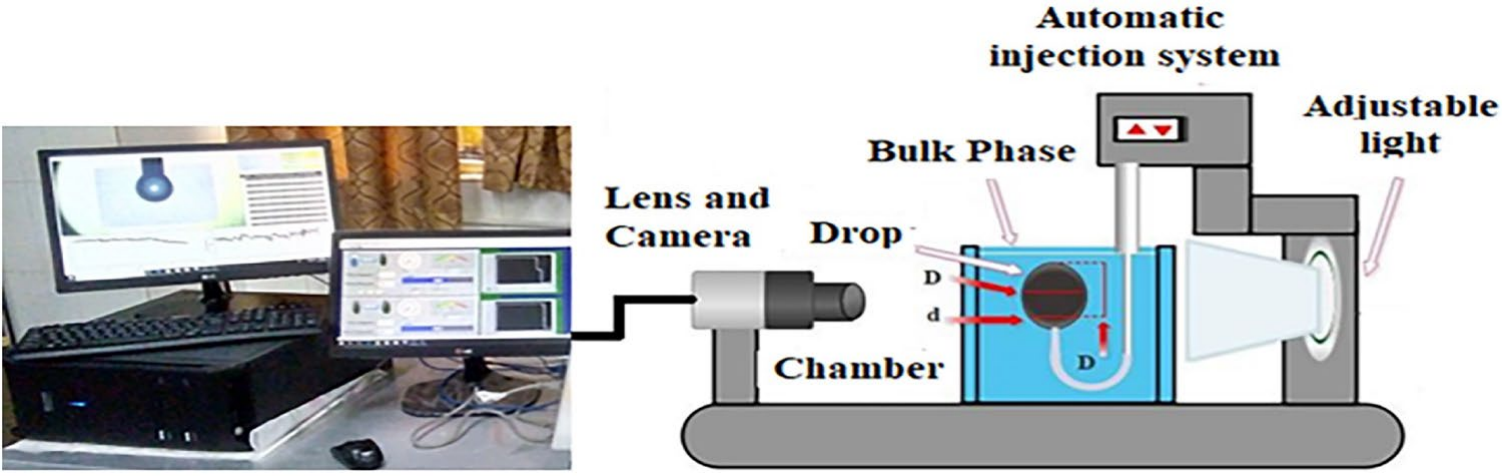
IFT and contact angle measurements equipment.

Besides, the neutral crude oil (crude oil I) has a TAN of 0.6 mg KOH = g oil with moderate contents of asphaltene and resin (6.8 and 7.9% wt%, respectively). Besides the acidity analysis, the Fourier transform infrared spectroscopy (FT-IR spectroscopy) using FT-IR equipment (WQF-510A, China) for the used sample crude oils was performed to find the functional groups of three crude oils shown (see Fig. 1). A close look into the depicted results in Fig. 1 revealed that the used crude oil I and crude oil II (acidic and neutral types) were the same considering the reported peaks in Fig. 2 and the FT-IR spectroscopy of the third crude oil (basic crude oil) was different from these two crude oils. One of the main criteria that directly affect the FT-IR spectroscopy is the C–H bonds seem similar for crude oil I and II. Besides, since the IR spectroscopy of all the examined crude oils is quite the same with some deviations in some peaks, it can be concluded that the used crude oil is quite the same in the basic functional groups. The point that must be clarified is that although they are similar in the FT-IR spectroscopy peaks; it is incorrect to assume similar structure or even composition (see Table 2) for these crude oils. Further investigations into the FT-IR spectroscopy results showed that the main functional groups of both crude oils comprise sulfoxide, sulfone, and carbonyl functions.

**Table 2:** The effect of IL on the IFT of the solutions under different pH values and crude oil types.

IL concentration (ppm)	IFT value (mN/m)								
	pH 3.5			pH 7			pH 11		
	Crude oil I	Crude oil II	Crude oil III	Crude oil I	Crude oil II	Crude oil III	Crude oil I	Crude oil II	Crude oil III
0	14.7	26.1	12.5	27.5	19.5	24.5	19.5	3.1	24.1
100	6.3	12.3	4.6	15.4	9.3	13.8	8.7	0.95	14.7
200	3.3	6.7	2.2	11.6	7.1	7.1	3.9	0.61	6.7
400	1.2	4.3	0.97	7.3	5.9	4.6	1.8	0.29	3.9
600	0.86	2.9	0.68	5.1	4.6	3.2	1.2	0.21	2.1
800	0.64	1.6	0.54	3.9	3.4	1.7	0.87	0.16	1.4
1000	0.55	1.1	0.38	2.4	2.7	0.93	0.66	0.08	0.98

### IFT measurement

Among the different IFT measurement methods, the pendant drop technique is one of the highly used methods due to its acceptable level of accuracy^61^ (Fanavari Atiyeh Pouyandegan Exir Co., Arak, Iran) (see Fig. 2).

The pendant drop method includes two individual but interconnected sections of drop suspension and image processing and image capturing sections. The first section which is purely mechanical section includes an automatic dosing system that injects a proper volume of a drop at the tip of the capillary nozzle where the drop forms to analyze its shape. The automatic injection section includes a glass syringe (Hamilton, USA) with a volume of 500 µL and an attached stainless U-shape flat-end needle with different sizes usually 500 µm.

The U-shape needle was used since the density of the crude oil phase was lower than the aqueous solution which means the oil drop would be suspended upward at the tip of the nozzle. On the other side, the aqueous solution or bulk phase was settled in an aquarium made of stainless steel and quartz glasses to provide a transparent view for the image-capturing section including a camera and macro lens. After suspending the drop, the image of the drop must be transferred into the online software where the shape of the formed drop is analyzed to calculate the shape factor, small and large diameters, and then convert these parameters to IFT values using the following equation:

1 $$ \gamma = \frac{{\Delta \rho g D^{2} }}{H} $$

where Δρ is the drop and bulk phases density difference, g is the acceleration of gravity, and H is the shape-dependent parameter. In Eq. 1, the H value is depending on the shape factor value, i.e. S = d/D, where D is the equatorial diameter and d is the diameter at the distance D from the top of the drop^62–65^. The noteworthy point is that the maximum uncertainty of the IFT for the current investigation was about ± 0.2 mN m^−1^ which was obtained from at least three independent measurements performed for each data point.

Since the pendant drop method has its own limitations for IFT measurement especially for the values below 0.5 mN/m, the spinning-drop method proposed by Vonnegut^65^ was used to calculate the IFT values below 0.5 mN/m according to below Eq. 2:

2 $$\upgamma \, = \,\left( {\Delta {\uprho  \omega }^{2} {{\text{R}}}^{3} } \right)/4 $$

where γ is the IFT in mN/m, R is the radius of the drop at the equator (E) with a unit of m, and ω is the angular velocity in rad/s, and ∆ρ is the drop and bulk density difference. A brief description of the used procedure is as follows. In the first place, the bulk phase was injected into the main chamber using a glass syringe with a volume of 10 cc to fill the bulk camber with a maximum volume of 2 cc. After that, the Hamilton syringe with a volume of 20 µl was used to inject a proper volume of a drop (2 µl) into the bulk phase using a long-size flat-end stainless steel needle since the drop must be injected in the middle of the bulk phase. After that, the rotational speed was increased not more than 3000 rpm to reach the criterion of Length/Diameter > 4 where the measurements are valid for the spinning drop method. As the drop was elongated to a proper length and diameter, the diameter was recorded using a camera and macro lens which dispatches the images to an online software where the IFT can be calculated using the measured parameters.

In other words, this formula is valid within 0.1% if the length of the drop exceeds 4 times its diameter. In practice, a more elongated drop is used which looks like a cylinder although the hemisphere at the tip of the drop does not have the same radius as the cylinder at the center. This is because of the centrifugal acceleration variation but increases with the distance from the axis. ∆ρ is the difference between densities of bulk and drop phases with a unit of kg m^−3^, and ω is the angular velocity with a unit of rad/s. The point is that according to the above formula, low tension will be associated with a small radius, i.e., elongated drops and slow rotational velocity, whereas high tension would require a high rotational velocity and the drop might not be elongated enough to fall in the Vonegut’s formula case. This method is thus appropriate to measure low tensions, typically below 1 mN/m, and down to ultralow values (µN/m or less) found in surfactant-oil–water systems containing microemulsions.

### Core flooding experiment (forced imbibition test)

**Figure 3:**
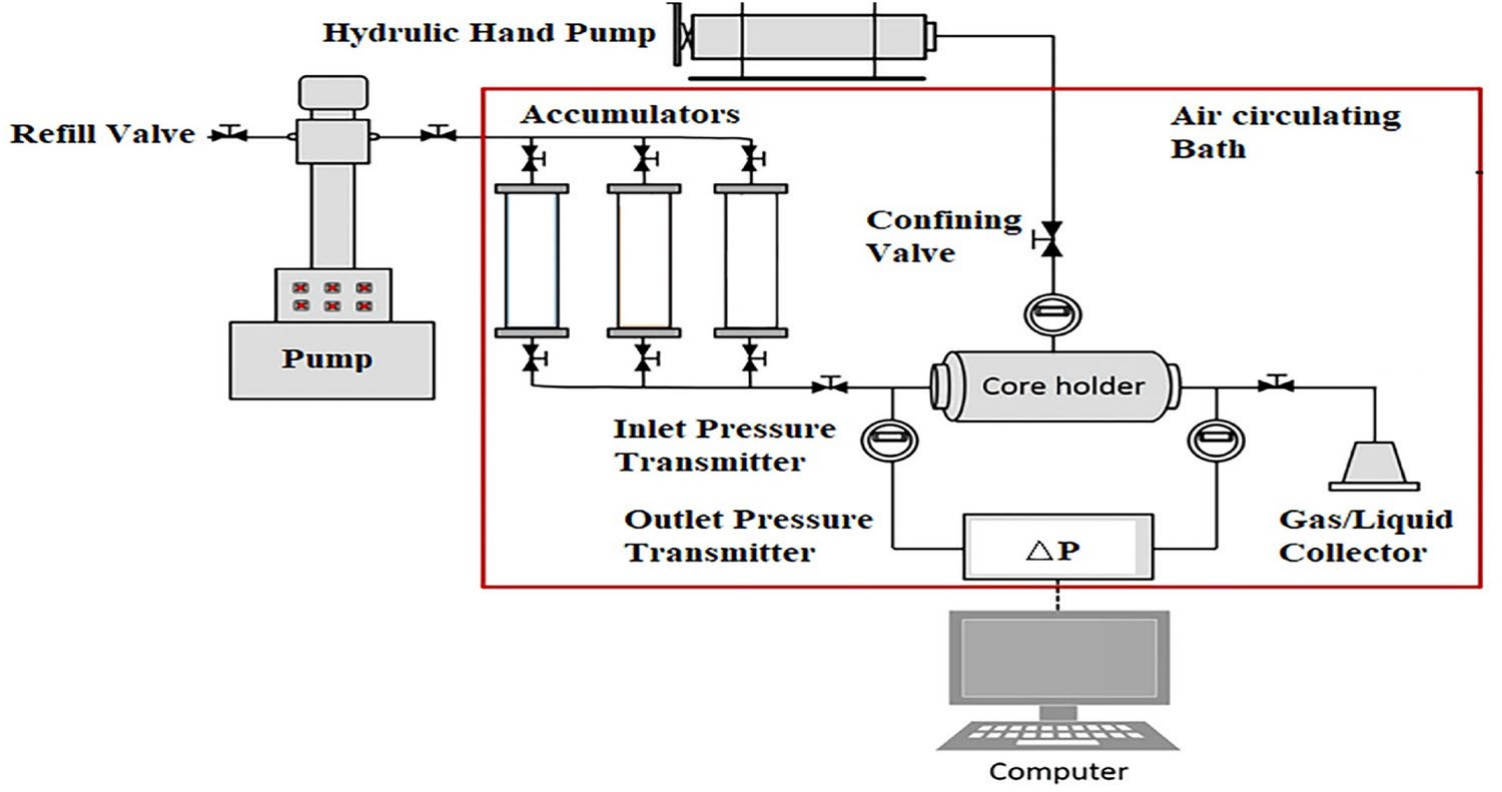
The schamtic of core flooding equipment.

The required core flooding experiments schematically introduced in Fig. 3 were performed using a core flooding equipment rated for maximum working temperature and pressure of 150 °C and 600 bar, respectively (APEX Technologies Co., Arak, Iran).

This apparatus mainly consists of several parts and the most important sections are accumulators, core holder, data acquisition system, high-pressure injection pump, air circulating heating bath, and gas back pressure regulator. The accumulators are used to inject the fluids into the core holder using a high-pressure injection pump and the core holder is used to keep the core plugs under a condition in which the injected fluid does not bypass the core due to the presence of confining pressure which provided using a manual high-pressure hand pump.

In this apparatus, the desired fluids are injected with a flow rate of mostly between 0.1 to 0.6 cc/min to mimic the laminar flow in the reservoir (fluids flow in the reservoir is about 1 ft/day which is close to 0.3 cc/min). The point is that this apparatus utilizes a gas back pressure regulator (GBPR) which provides the chance of injecting the fluids, especially the gases and live oil under a reservoir pressure. In detail, it is required to increase the injection pressure to the values higher than the pressure inserted into the doom of the GBPR to take out the injected fluid into the core holder. So, the current apparatus can be used as a way to perform the different injection patterns and inject different solutions under different pressures and temperatures using a proper injection sequence. In light of these facts, the following injection sequence can be used to mimic the tertiary oil recovery using dead oil with different chemical solutions:

- Porosity and permeability measurements
- Fresh core saturation with the desired saline solution
- Crude oil injection with a flow rate of 0.3 cc/min to reach the irreducible water saturation (S_wirr_).
- Formation brine injection with a flow rate of 0.3 cc/min to mimic the water flooding (secondary flooding) stage
- Tertiary oil recovery stage using injection of optimum chemical formulation with a flow rate of 0.3 cc/min and selected pore volumes (PVs) of chemical solution.
- Injection of several PVs of saline solution to push the injected PVs of the optimum chemical formulation to reach the ultimate oil recovery (the point no oil was produces similar to the secondary oil recovery stage). It is possible to change the injected volume of the chemical solution and flow rate to investigate the impact of these two operating parameters if they are considered the target of the study.

### Adsorption

In the current investigation, the adsorption of the IL as a surfactant was examined via a dynamic adsorption procedure instead of batch adsorption experiments usually performed for the surfactant and polymer adsorption reported in the literature^66–70^. The dynamic adsorption experiments were conducted using the method previously reported by Hezave et al.^55^ under room temperature. In detail, in the first place, the required chemical solutions were prepared with known concentrations, and the values were recorded. After that, several pore volumes (PVs) of each chemical formulation (maximum PVs of 20) with known concentration were injected into the selected cores using the core flooding apparatus. The core flooding system was rated for pressure and temperature of 600 bar and 150 °C with three different accumulators and one Hassler-type core holder.

For each adsorption test, the concentration of IL was set at 2000 ppm (above the critical micelle concentration (CMC) to ensure we can achieve the ultimate effect of IL in the system). After preparing the solution, the aqueous solution was injected with a flow rate of 0.3 cc/min into two prepared cores. The first type of prepared core used for direct injection of the aqueous solution was the fresh and dried core without any oil and formation brine in its structure. The aqueous solution was injected into the core for several pore volumes to a point that the concentration of the injected solution got close to the produced solution (in some cases it is impossible to reach the 100 similarity between the injected and produced aqueous solution due to monolayer and equilibrium adsorption phenomenon). In the second scenario, the core was flooded with several PVs of formation brine to reach full saturation with formation brine. After that, the saturated core with formation brine was flooded using selected crude oils to reach a point of no formation brine being produced. At this point, the core reached irreducible water saturation means the core is prepared for aqueous solution injection. For both scenarios using different pH values and different crude oils, the effluent fractions were collected and analyzed using UV spectrophotometry (Thermo Scientific GENESYS 10 UV Visible Spectrophotometer, USA). Since the surfactant has a maximum absorbance at 212 nm with a linear response up to 200 ppm, the effluents were well diluted to ensure reaching a concentration that the linear calibration curve is valid.

## Result and discussion

### Effect of chloride salts and pH on the IFT of different crude oils

**Figure 4:**
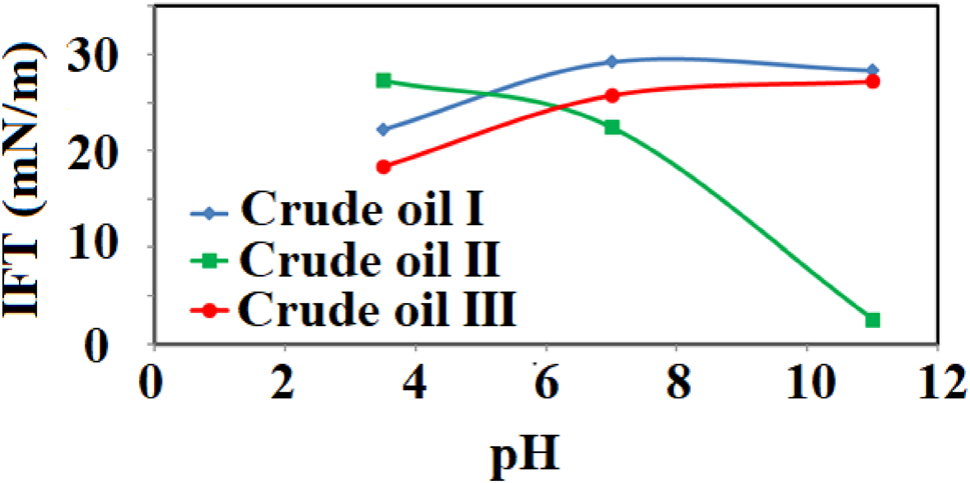
The IFT variation as function different crude oils.

In the first stage of this investigation, the effect of chloride salts was examined on the IFT of the different crude oils (see Fig. 4). In this way, the aqueous solutions were prepared using 5000 ppm of each salt to meet the low salinity condition and the pH value was changed between 3.5 to11 while three different crude oils of neutral (crude oil I), acidic crude oil (crude oil II), and basic crude oil (crude oil III) were used.

As shown in Fig. 4, the crude oils with different natures led to different IFT behaviors using distilled water (DW). A close look into Fig. 4 revealed that increasing the pH has a reducing effect on the IFT value of acidic crude oil (crude oil II). The reason for this trend can be directly correlated to the saponification process which acts as the primary in-situ surfactant and IFT reducer chemical. According to these findings, the IFT reduction for the acidic crude oil can be correlated to two different reasons; the first one is the saponification process and in-situ formation of surfactant leading to an IFT reduction and the reorientation of natural surfactants at the interface. The impact of in-situ surfactant formation and asphaltene/resin effects on the IFT reduction can be described according to the following phenomenon. As the in-situ surfactant forms in the solution, they can be moved toward the fluid interface and reduce the IFT. On the other side, since the acidic crude oil is comprised of a high content of asphaltene and resin with hetero atoms, repulsive forces between the molecules of these two fractions may move them far from each other consequently leading to IFT enhancement. In light of this fact, any reduction in the repulsive forces provides a chance of higher concentration of these molecules in the interface leading to higher IFT reduction due to the surface activity nature of asphaltene and resin fractions. In other words, since the asphaltene and resin fractions are similar to the natural surfactants with IFT reduction capability, any attempt (in the current work is the in-situ surfactant production) which moves these fractions toward the interface reduces the IFT of the system. The in-situ surfactant molecules can be oriented in the bulky structure of the asphaltene and resin fractions to neutralize and reduce the extensive repulsive forces of these fractions means the possibility of a higher number of the natural surface active molecules to be packed in the interface, and lower IFT values.

Further investigation in Fig. 4 revealed that the pH causes a reverse effect on the IFT of the neutral and basic crude oils. The depicted results showed that for the low pH values, the IFT is lower than the conditions where pH is neutral or basic. The reason for this observed trend is directly correlated to two reasons; the first one is the boosted repulsive forces because of the basic nature of the crude oil and the OH^−^ ions. However, the second reason is attributed to higher repulsive forces between the asphaltene and resin fractions since the asphaltene and resin molecules have a negative nature. Because of these similar negative charges, the asphaltene and resin molecules move far from each other and far from the interface to reduce these repulsive forces leading to higher IFT values. The other point is that low pH values concomitant with acidic crude oil reduce the ionization of crude oil components and increase the positive repulsive forces in the solution leading to higher IFT values.

Moreover, as the pH for the neutral and basic crude oil systems increased, the IFT was also increased. This observed trend is directly correlated to the negative effect of OH^−^ ions in the solution which can increase the repulsive forces between different molecules and ions in the interface. So, an increase in the repulsive forces puts the molecules far from each other and prevents the packing of the ions in the interface which is essential to reduce the IFT.

**Figure 5:**
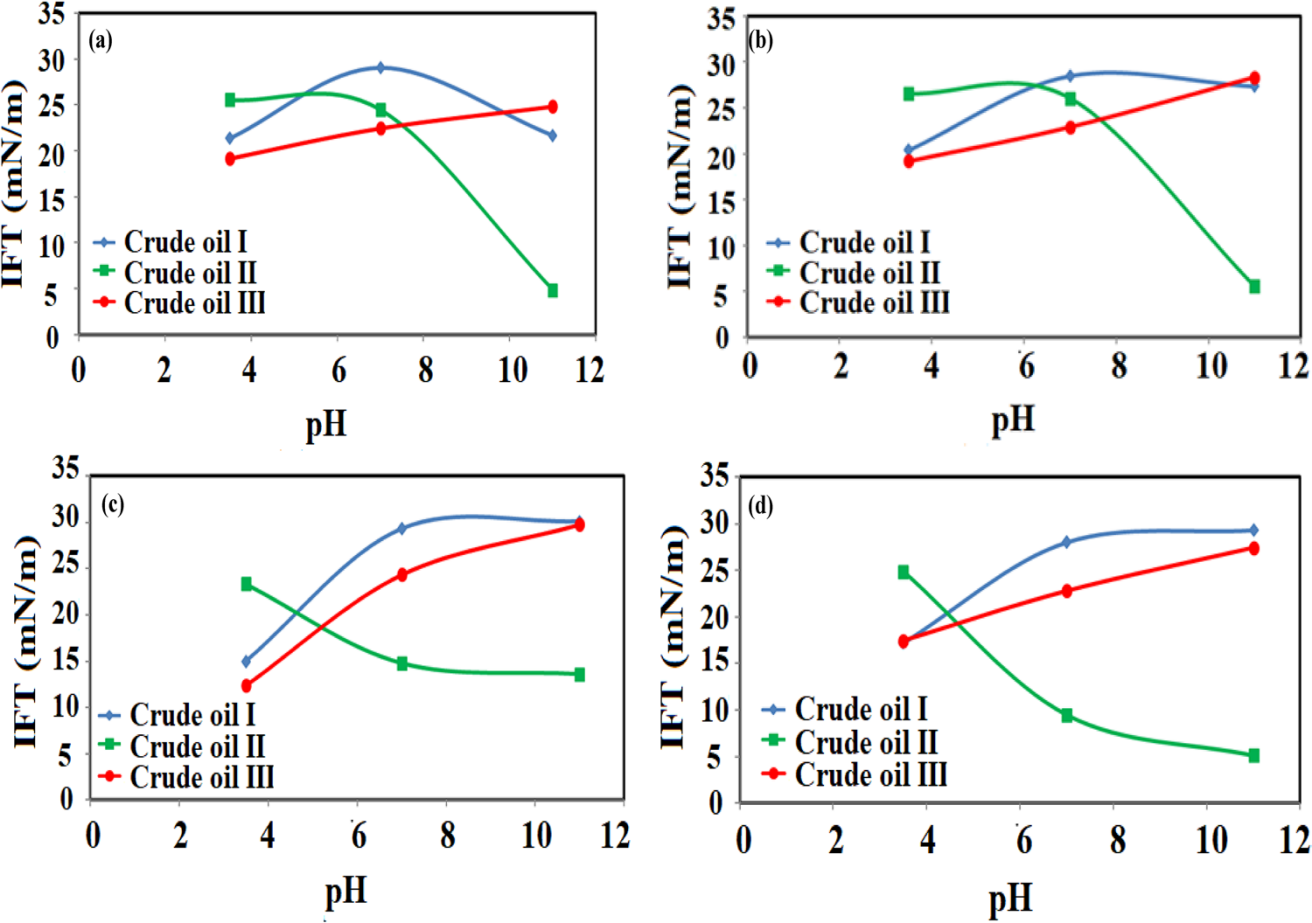
IFT variation as a function of pH, (**a**) NaCl, (**b**) KCl, (**c**) CaCl_2_, (**d**) MgCl_2_.

In the second stage of this section, the effect of chloride salts including NaCl, KCl, MgCl_2_, and CaCl_2_, and pH values of 3.5, 7, and 11 was examined on the IFT of three different crude oils. The point is that for the basic and neutral crude oils, the effect of chloride salts is rather the same although they have a general reducing effect on the equilibrium IFT (EIFT). In detail, comparing the measured IFT values for the neutral and basic crude oils in the presence of chloride salts revealed that the IFT was reduced compared with the IFT values obtained using DW while no significant change in IFT variation pattern was observed. In contrast, the results revealed that the effect of salts on the IFT of acidic crude oil was rather different. The results in Fig. 5 showed that the IFT variation pattern without its reduction was similar to the results obtained for DW, but in the case of divalent ion salts; the IFT variation trend was different. In detail, for the monovalent ions, the IFT sharply reduces as the pH value increases to a value higher than 7, and for the case of divalent ions, the IFT sharply reduces as the pH value increases from 3.5 to 7 and then experiences a slight reduction as the pH was increased from 7 to 11. This observed trend can be correlated to two different reasons for forming in-situ surfactants for the acidic crude oil and the salting-in effect for all examined crude oils. The salting-out and salting-in effects are one of the most important and well-known mechanisms for the low salinity systems dealing with salts with a specific concentration lower than 5000 ppm. In detail, in the first step, any formation of in-situ surfactant and its distribution into the interface has an undeniable effect on the IFT reduction which is highly effective for acidic crude oil because of its nature compared with the basic and neutral crude oils. Besides, the presence of salts can manipulate the surface active component distribution existing in the oil phase (such as asphaltene and resin fractions) into the aqueous phase or the oil phase^71^. The salting-in effect which is the opposite phenomenon of the salting-out effect (mostly occurs due to high salinity conditions) is responsible for polar organic species dissolution in water which directly affects the IFT reduction^72^. So, it seems that in the current investigation, the examined systems (used three crude oils) experience hydrogen bonds because of salts e.g., Na^+^, K^+^, Ca^2+^, and Mg^2+^, and organic compounds enhance the solubility of organic molecules in the water phase consequently leading to IFT reduction which is in contrast to the general beliefs.

In brief, the salting-in effect can boost the diffusion of surface active components from the bulk solution to the interface under low salt concentrations while increasing the salts concentrations to values higher than the low salinity threshold (5000 ppm) can activate a reverse mechanism which is the salting-out effect that retard the dissolution of petroleum hydrocarbon species in the aqueous which means higher equilibrium IFT values^73–77^.

Investigating this mechanism is highly important since it is expected that the presence of salts has an increasing effect on the IFT for the hydrocarbon and water systems (without natural surfactants such as asphaltene and resin, which naturally exist in crude oil)^78–81^.

In contrast to the increasing effect of salts on the IFT of oil/water, several researchers such as Alotaibi et al.^82^ and Serrano-Saldana et al.^83^ reported a reducing IFT trend for the studied systems such as dodecane + brine and dodecane + NaCl aqueous solutions, respectively. They claimed that this observed trend is directly correlated to the salting-in effect leading to transferring the active components of the crude oil toward the aqueous phase means a lower IFT value. To sum up, considering the salting-in influence and the formation of in-situ surfactants due to the saponification process, the in-situ surfactant can accelerate the salting-in effect for higher IFT reduction. In other words, monovalent salts and in-situ formation can amplify and strengthen each other to sharply reduce the IFT for pH values above 7 (pH value of 11). But for MCl_2_ and CaCl_2_, the situation is different and it seems that the presence of divalent salts is capable to reduce the IFT value of the systems to its minimum value without the saponification process and in-situ formation although the saponification process (for the pH values higher than 7) has a slight effect for IFT reduction. The reason behind this conclusion is that the IFT experience a sharp reduction for the pH values lower than 7 in the presence of divalent salts while increasing pH from 7 to 11 which undoubtedly leads to the saponification process, has a moderate effect on the IFT reduction and reach to the ultimate IFT value. So, it seems that the presence of divalent salts provides rather an ultimate effect for IFT reduction and the presence of alkali which is produced during the saponification processes introduces its operational effect not only for IFT reduction but also they can act as sacrifice to prevent surfactant adsorption if the surfactant existed in the solution.

The other point is that since the divalent salts have higher surface charges than the monovalent salts, a dominant salting-in effect is achieved causing better bonding between the active components of the crude oil and the divalent salts moving them toward the aqueous phase. So, the influence of divalent salts on the IFT reduction in light of the salting-in effect is more evident than in the system that deals with monovalent salts.

### Effects of sulfate salts and pH value on the interfacial tension reduction

**Figure 6:**
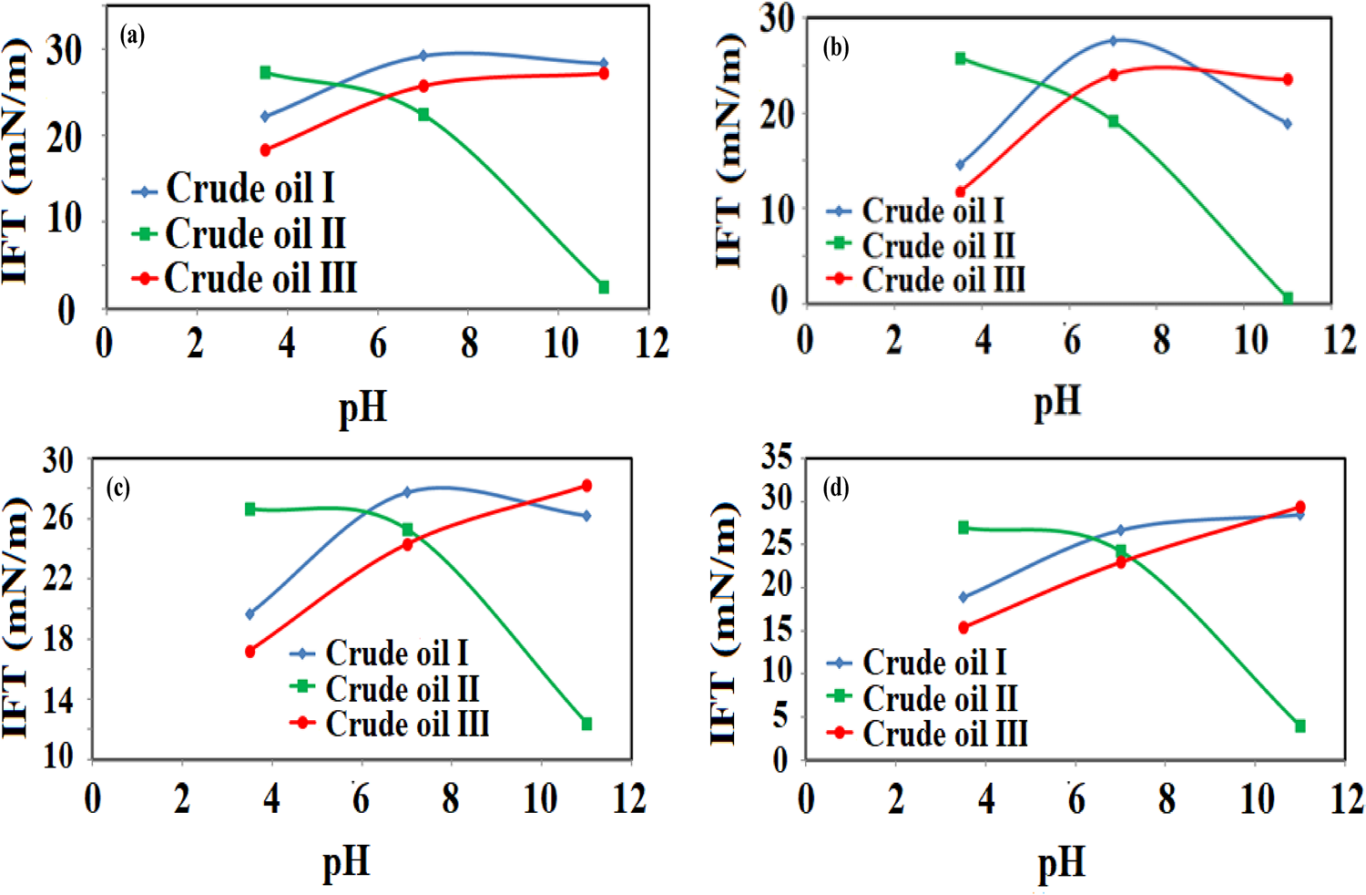
The IFT variation as a function of pH, (**a**) DW, (**b**) Na_2_SO_4_, CaSO_4_, (**d**) MgSO_4_.

In the next stage of this investigation, the effects of sulfate-based salts were investigated using different crude oils (see Fig. 6) by changing the pH in the range of 3.5 to 11. The result revealed that the presence of divalent salts has a similar effect on the IFT compared with the monovalent salts although the overall effect was lower IFT values, especially for the acidic crude oil under a high pH value of 11. Similar to the results obtained for the monovalent salts, an increase in pH for all the examined sulfate salts led to a considerable reduction in IFT value especially for pH value of 11 in which the saponification process reached to its ultimate point. But, in contrast to the results obtained for the divalent and monovalent chloride salts, the presence of different sulfate salts has no superiority to each other which showed the complementary effect of the saponification process to increase the IFT reduction rate. In detail, the results revealed that the presence of salts (under a pH value of 3.5) compared with the systems dealing with DW causes a reduction in IFT value. Besides, increasing the pH from 3.5 to 7 led to a further reduction in IFT although this reduction was insignificant. But, as the pH was increased from 7 to 11, a sharp reduction in IFT was observed showing the dominancy of the saponification process on the IFT reduction compared with the presence of salts. In contrast to the results obtained for the acidic crude oil, the results revealed that an increase in pH for all of the examined salts in contact with the neutral and basic crude oil negatively affects the IFT due to an increase in the repulsive forces that move the active molecules to a far distance from the each other and interface consequently leading to IFT increase and eliminate the reducing effect of salts on the IFT.

**Figure 7:**
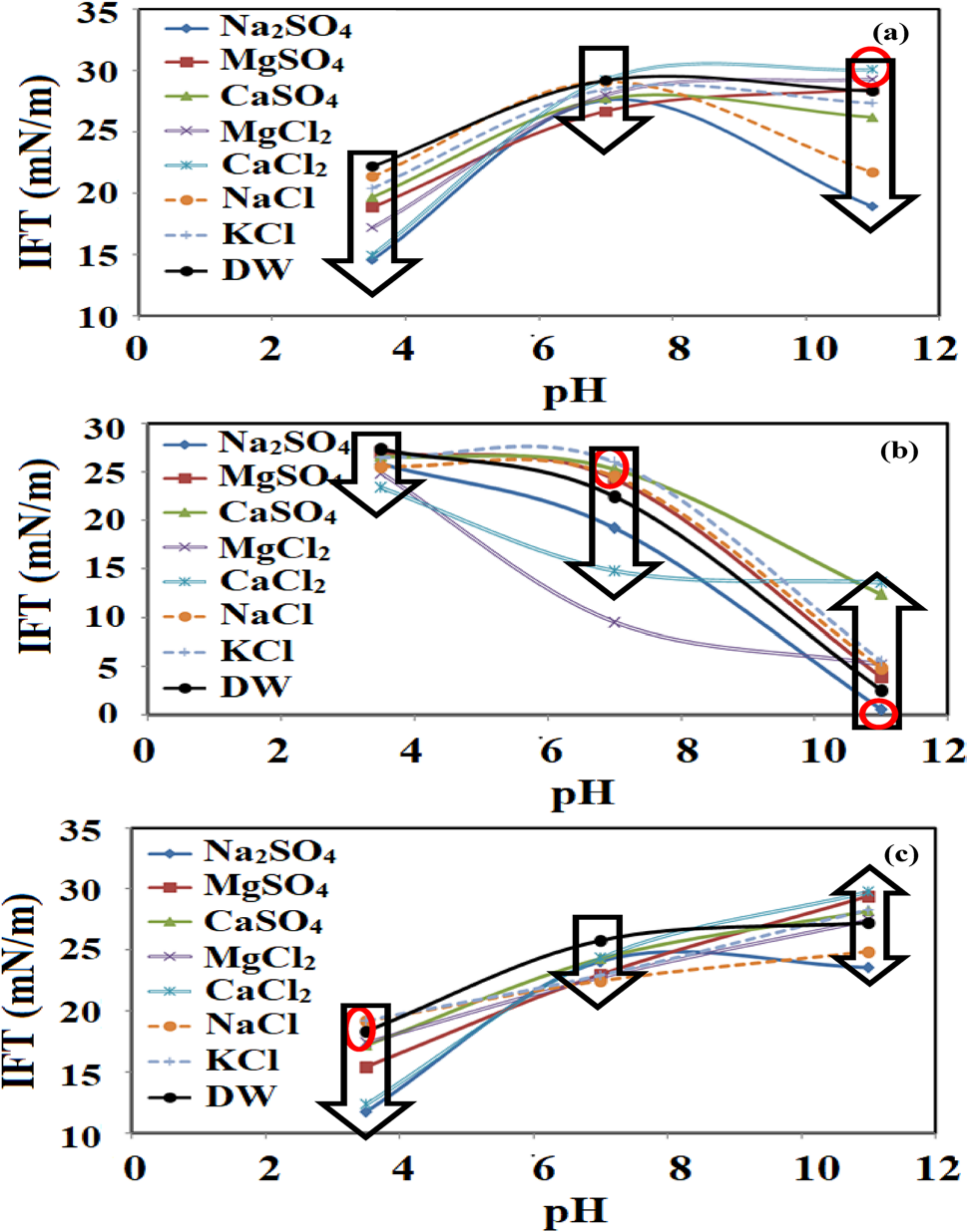
IFT variation as a function of pH, (**a**) Crude oil I, (**b**) Crude oil II, (**c**) Crude oil III.

Among the examined sulfate salts, it seems that the highest impact on the ultimate IFT value and IFT trend belongs to Na_2_SO_4_. In detail, the presence of Na_2_SO_4_ led to the appearance of a point around pH of 7 in which the IFT experienced a shifting pattern except for the acidic crude oil. The depicted results revealed that in the case of using Na_2_SO_4_ for both neutral and basic crude oils, an increase in the pH value led to an increase in the IFT value while the IFT was reduced if the pH was further increased from 7 to 11. According to these findings, the presence of Na_2_SO_4_ in the aqueous solution significantly affects the IFT reduction for all the examined crude oils under basic conditions (pH value of 11). For more clarification and easier comparison, the obtained results were depicted together for each crude oil in Fig. 7. A close look into Fig. 7a revealed that for the neutral crude oil, the presence of salts regardless of their type causes a reduction in IFT value for pH value of 3.5 and 7 while an increase in the pH to values of 11 change the reducing trend for CaCl_2_ and MgCl_2_ while the other salts leading to IFT reduction similar to the results observed for pH values of 3.5 and 7. These observed trends were also observed for the system dealing with basic crude oil only with this difference that for the pH value of 11, the presence of only NaCl and Na_2_SO_4_ provide a positive effect for IFT reduction and the other salts leading to higher IFT value compared with the DW solution.

The reason for this observed trend can be correlated to the fact that as the pH increases for basic crude oil, the presence of OH^−^ provides more repulsive forces between different components of the system that are amplified by salts except NaCl and Na_2_SO_4_ which can compensate the negative effect of repulsive force and keep the IFT value lower than the system dealing with DW. In contrast to the basic and neutral crude oils, the acidic crude oil extensively was affected toward lower IFT values for all of the examined situations of pH and salts due to two different mechanisms of salting-in effect and saponification process strengthening and amplifying each other.

### Effect of ILs on the IFT reduction of optimum chemical formulation

In the current section, the effect of IL namely 3-dodecyl 3-methyl imidazolium chloride ([C_12_mim][Cl]) was investigated on the IFT reduction of optimum formulation of Na_2_SO_4_ leading to the most desired IFT values in most of the cases using three different crude oils and different pH values. In this way, the concentration of the IL was changed between 100 to 1500 ppm to find the critical micelle concentration (CMC) in the first stage and then find the optimum concentration leading to the lowest IFT value for all of the examined systems (crude oil and different pH values) (see Table 2). The performed IFT measurements using different pH values and crude oil types revealed that in the presence of IL especially for the concentrations higher than 400 ppm that can be considered as the critical micelle concentration (CMC) for all the examined cases, the IFT reduction was independent of the pH and crude oil type. In detail, for all the crude oil types and pH values, as the IL concentration was increased to 400 ppm from 100 ppm, an extensive reduction in IFT was observed for all the cases although further reduction in IFT was also observed for the concentration higher than 400 ppm. But the point is that, although all the measured IFT values were at the same level, the minimum IFT value was observed for the IL concentration of 1500 ppm and the acidic crude oil under basic conditions (pH of 11) with an IFT value of 0.08 mN/m. The possible reason behind this observed minimum IFT value can be the synergy between the in-situ surfactant produced during the saponification process and the presence of IL .

According to the results, it is possible to select concentrations higher than 400 ppm as the optimum concentration for the minimum IFT value and higher oil recovery. However, selecting the best concentration of IL (400 ppm or higher concentration) is impossible if no adsorption data are available. In detail, since the adsorption of IL on the rock surface under different operating conditions and crude oil type is a crucial parameter for proper decision, the next stage of this investigation was designed and performed to measure the adsorption of the IL on the rock surface under different conditions.

### Measuring the adsorption of IL on the rock surface

In the current phase of the investigation, the aqueous solutions prepared using the Na_2_SO_4_ with a concentration of 5000 ppm and IL with a concentration of 2000 ppm well above the CMC value (400 ppm) were used to measure the adsorption of IL under different pH values and using different types of crude oils (see Table 3).

**Table 3:** The ILs adsorption under different operating conditions.

No	pH 3.5			pH 7			pH 11		
Oil type	Acidic	Neutral	Basic	Acidic	Neutral	Basic	Acidic	Neutral	Basic
Adsorption (mg IL/mg rock	0.83	0.69	0.74	0.75	0.74	1.0	0.42	0.80	1.11

Investigating surfactant adsorption is highly important since about half of the chemical injection cost is related to the surfactant usage. So, any unusual adsorption of the surfactants during the EOR processes renders the process towards an uneconomic situation. In general, the adsorption of surfactants adsorbs onto solid surfaces in monomer form rather than as micelles under different mechanisms. These mechanisms are ion exchange, ion association, hydrophobic bonding, adsorption by the polarization of π electrons, and adsorption by dispersion forces^84^. Usually, the surfactant adsorption on the rock surface is correlated to surfactant type, surfactant concentration, surfactant equivalent weight, ionic strength, pH, salinity, and temperature^85^. These factors can also influence the dissolution behavior of minerals, and therefore, it will cause significant changes in the adsorption of surfactants into the rock surface^86^.

Among these parameters, pH is one of the most crucial operating parameters that can be set at a desired value to reach the minimum surfactant adsorption. So, the effect of pH at different values on the adsorption of IL surfactant in the presence of different crude oils of acidic, neutral, and basic was investigated in the current section. In light of this fact, 12 different dynamic surfactant adsorption measurements were performed using two different approaches. The first approach (AP#1) is based on the injection of a prepared solution into fresh and dried cores (3 measurements for three pH values of 3.5, 7, and 11). However, the second approach (AP#2) (9 different adsorption measurements) was used to measure the adsorption of surfactant by injecting the prepared solutions into the cores that were flooded and saturated by saline water (optimum chemical formulation without IL) in the first place. After that, the saturated cores were flooded by oil to reach the irreducible water saturation followed by saline water injection to reach the residual oil saturation (S_or_). These two approaches were selected since the first approach provides a good insight regarding the direct adsorption of the surfactant into the core surface in the absence of crude oil and the second approach provides a good insight respecting the possible partitioning and adsorption of the surfactant into the saline water/crude oil and rock surface together.

**Figure 8:**
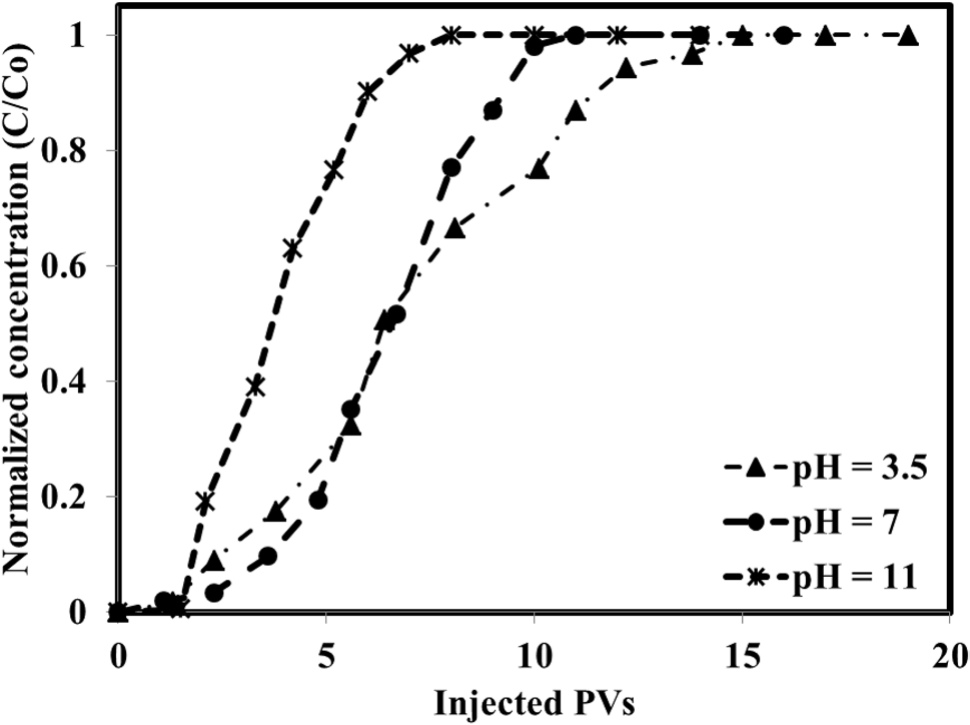
The IL adsorption into the fresh rock surface and structure under different pH values.

The first series of the adsorption measurements were performed using fresh and dried cores and optimum chemical formulation (5000 ppm Na_2_SO_4_ and 2000 ppm of IL that is well above its CMC value) under different pH values of 3.5, 7, and 11. The measurements which are depicted in Fig. 8 revealed that as the pH of the system increases, the required PVs to reach a constant concentration in the effluent (well close to the injected solution) reduce to about 8 PVs while for the pH values of neutral and acidic, PVs of about 11 and 14 PVs, respectively were required. The calculated adsorption values revealed that an increase in the pH leads to a lower rate of adsorption but a higher amount of adsorption. In detail, the calculated adsorption values showed that the adsorption was increased from 0.68 mg IL/g rock to 1.30 mg IL/g rock as the pH was increased from 3.5 (acidic condition) to a pH value of 11 (basic). The main reason behind this observed trend can be correlated to this phenomenon that the used surfactant has a positive charge and the used carbonate rock also has a positive surface charge due to calcite and the other positive surface minerals. In this way, for the acidic conditions and even neutral conditions, the IL adsorption is lower than the adsorption observed for a pH value of 11 due to higher repulsive forces. In detail, under acidic and neutral conditions, the repulsive forces that existed between the rock surface and IL (both are positively charged) prevent the interactions and adsorption of IL into the rock surface consequently reducing the adsorption of the IL to values of 0.68 and 1.08 mg IL/g rock for pH values of 3.5 and 7, respectively. In detail, surface charges that exist on the rock surface as well as the surfactant have a direct and undeniable impact on the surfactant adsorption. Generally, anionic surfactants are usually negative where cationic surfactants (here the used IL is cationic) possess a positive charge and they are by default attracted to positively charged surfaces and negatively charged surfaces, respectively. On the other side, the formation brine composition and pH directly affect the surface charges. In general, if the effect of brine composition is ignored, anionic surfactants usually tend to be adsorbed on the carbonates (positively charged surface) at the neutral condition (pH 7) due to the existence of positive charges on the rock surface. However, the cationic surfactants with positive surface charges are likely to be adsorbed on the sandstone rock surface with negative charges^28,84^. Considering this hypothesis and mining into the previously published works, it seems that the main mechanism governing IL adsorption pattern is van der Waals’ interactions and electrostatic interaction^87^.

But, the point is that the adsorption phenomenon is a complex one especially for the ILs due to their unique structure which is probably a combination of different phenomena including electrostatic interactions, hydrogen bonding, chemical interactions, covalent bonding, nonpolar interactions, and desolvation of the adsorbate moieties. In the light of this complex combination, it is possible to categorize the adsorption pattern into three stages. The first stage occurs due to the electrostatic interaction (mostly between rock surface and individual charged monomeric species). The second one is a linear variation in adsorption in increasing pattern that comes from the interaction between the previously adsorbed IL molecules and the new IL molecule that is going to be adsorbed (interaction between the hydrophobic chains of ILs). In the last stage, adsorption occurs because of the electrostatic attraction between the solid surface charge and IL species and hydrophobic interactions between the hydrocarbon chains. Finally, it is possible to consider further adsorption due to the electrically neutral surface which provides the chance of adsorption due to the chain–chain hydrophobic interactions alone^31^.

**Figure 9:**
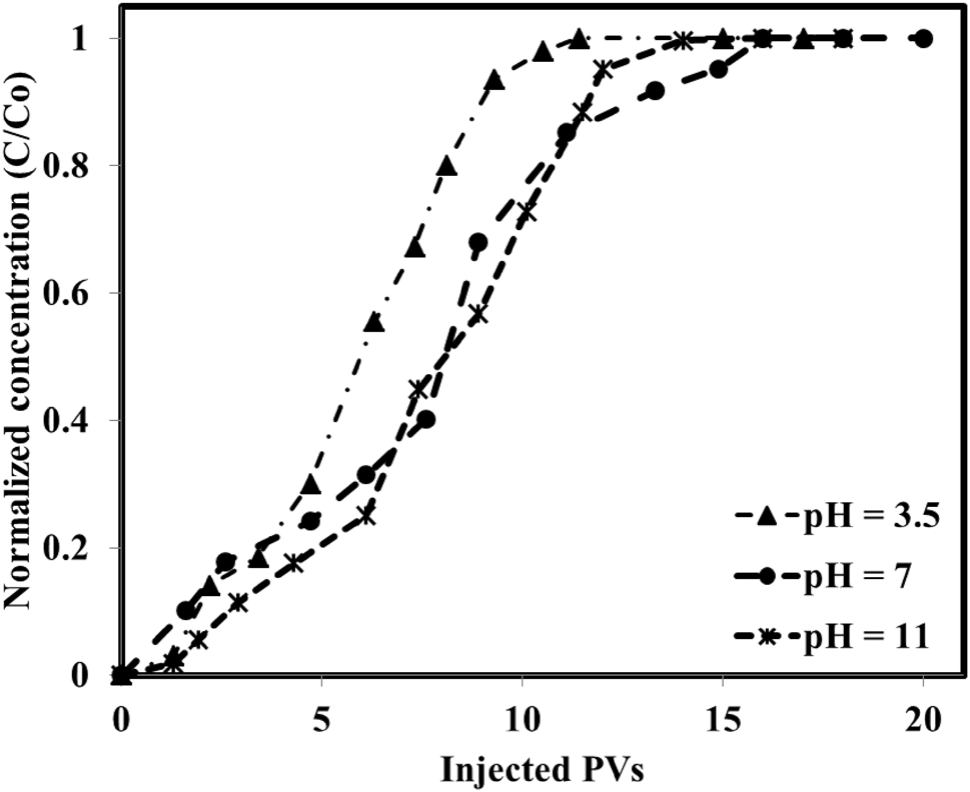
Effect of pH on the IL adsorption of basic crude oil/aqueous solution.

**Figure 10:**
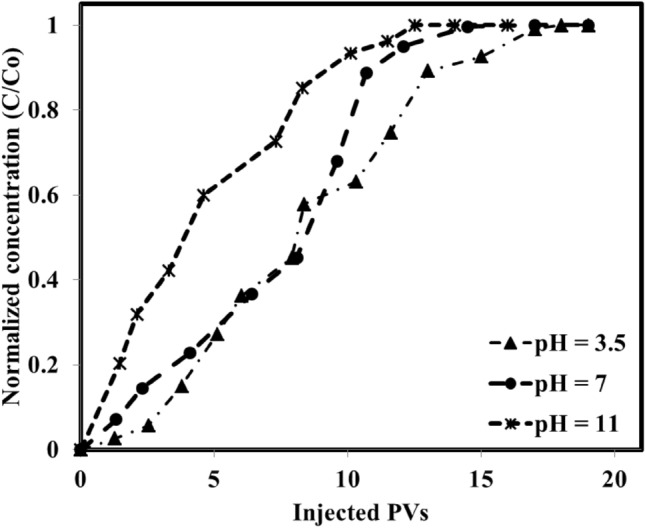
Effect of pH on the IL adsorption of neutral crude oil/aqueous solution.

Further investigations on the adsorption of the IL on the rock surface of the system dealing with basic crude oil/aqueous solution under different pH values revealed that as the pH was increased from 3.5 to 11, the adsorption was increased from 0.74 mg IL/g rock to 1.11 mg IL/g rock (see Fig. 9). The measurements revealed that although the higher IL adsorption under a higher pH value (11) was obtained, this is the neutral condition (pH 7) causes a lower rate of adsorption and larger PVs to reach the ultimate adsorption on the rock surface. This observed trend is due to the electrostatic forces between the aqueous solution, crude oil, and the rock surface. In detail, for pH of 3.5 and 11, the situation is so clear that the repulsive forces are at the minimum and maximum of their values due to the presence of H^+^ (acidic condition) and OH^−^ (basic condition) in the solution. So, the system achieves a fast equilibrium of adsorption with a lower number of injected PVs while for the neutral condition which has moderate electrostatic forces (repulsion and attraction), there are adsorption and desorption processes that move the system toward a lower rate of reaching equilibrium condition. Further adsorption measurements using the neutral crude oil under different pH values revealed that the adsorption value was changed between 0.69 mg IL/g rock to 0.8 mg IL/g rock as the pH was increased from 3.5 to 11 (see Fig. 10). According to these findings, one can conclude that the adsorption of neutral crude oil on the rock surface is a weak function of pH as it was changed from 0.69 mg IL/g rock to 0.8 mg IL/g rock. In other words, since the neutral crude oil nature is neither positive nor negative, the presence of OH^−^ and H^+^ in the solution which can manipulate the rock surface charge has an insignificant effect on the higher or lower adsorption of the IL on the rock surface.

**Figure 11:**
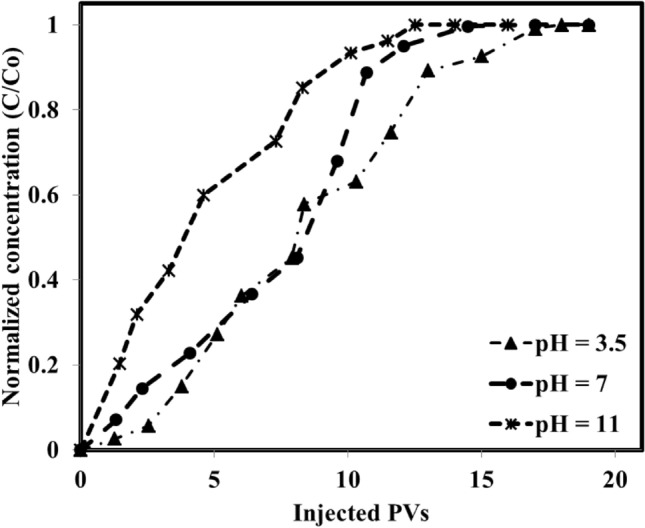
Effect of pH on the IL adsorption of acidic crude oil/aqueous solution.

In the last stage of this section, the IL adsorption on the rock surface dealing with acidic crude oil/aqueous solution was examined. The adsorption measurements revealed that as the pH was increased from 3.5 to 11, the adsorption was significantly reduced from 0.83 mg IL/g rock to 0.42 mg IL/g rock (see Fig. 11). The possible mechanism behind this observed trend is the saponification process concomitantly with rock surface charge modification from positive charge to negative charge due to presence of OH^−^ ions and the possible orientation of OH^−^ ions between the bulky structure of the ILs which can reduce the repulsive forces exist between the IL molecules in the interface. In detail, since the IL molecules have a bulky structure, the OH^−^ ions move into the bulky structure and orient in them in a way that the positive surface charges of these molecules experience a reduction. As a consequence of this reduction in surface charge, a larger number of IL molecules can be moved into the interface regardless of the liquid/liquid or liquid/rock interfaces. So, higher amounts of IL molecules are adsorbed on the liquid/liquid interface which has a reducing effect on the IFT values, and also higher adsorption on the rock surface affects the wettability of the rock surface. But, on the other side, the presence of OH^−^ ions and the organic acidic compounds of acidic crude oil provide the chance for the saponification process and production of in-situ surfactants in the solution known as the alkali. These alkali molecules have the potential to be firstly adsorbed in the rock surface in the presence of surfactant molecules. As the alkali is adsorbed on the rock surfaces, the available active sites for IL molecule adsorption experience an extensive reduction due to the adsorption of the alkali molecules in the first stage. As a result, the adsorption of the surfactant molecules experiences a reduction in favor of the chemical EOR flooding expense and efficiency.

On the other side, it is possible to correlate the impact of alkali for surfactant reduction to the pH enhancement because of alkali consequently reduces the number of positive sites available for the adsorption on the carbonate surface which means lower adsorption. For example, Pillai and Mandal^27^ reported that adsorption experienced an enhancement for [C_12_mim][BF_4_] as the alkali concentration was increased especially if it was increased to around 1.5%.

Moreover, they reported that the IL adsorption at the low concentration of alkali takes place because of the primary movement of the adsorption position at the interface to the top of the adsorption monolayer which has a direct impact on the changing the hydrophobicity of the surface to a certain extent consequently leading to higher adsorption. They also reported that as the alkali concentration was further increased to 1.5%, IL molecules arranged with a more compact combination in a monolayer pattern which has a polar head group packed and oriented toward the rock surface moving the rock surface toward the maximum hydrophobicity condition. The point is that a further increase in the alkali concentration leads to a reduction in the rock surface hydrophobicity in the light of lower IL adsorption due to the strong electrostatic repulsion between the mineral sites and IL molecules starting to stabilize the rock surface charge^88^.

In more detail, the application of alkali in surfactant flooding is usually utilized to produce in situ soap because a reaction occurs between the acidic contents of crude oil and alkali which reduces the required amount of surfactant during surfactant flooding which directly affects and lowers the operating costs and significant improvement in the profit of EOR projects. Moreover, the application of alkali is desired since it can reduce the adsorption of surfactant as pH is increased consequently increasing the surfactant stability^89^. But the point that must be carefully examined is that the impact of alkali in lowering the adsorption of surfactants is limited to situations where the salinity/hardness is in a reasonable range due to alkali sensitivity to Ca^2+^ and Mg^2+^ and probable precipitation of alkali^90,91^. In detail, the presence of multivalent ions like Na^2+^, Ca^2+^, and Mg^2+^ can be adsorbed on the negatively charged rock surfaces and may also reverse the surface charge where the cationic surfactants have lower adsorption on the rock surface^92,93^.

Considering this fact, if the surfactant slug with anionic surfactant is injected into the reservoir, surfactant loss occurs leading to low oil recovery. However, if the cationic surfactants imply in the solutions with these multivalent ions, lower IL precipitation and adsorption occur due to electrostatic repulsion^57^.

Moreover, the previously published works revealed that as the amount of divalent ions in the formation brine increases, the adsorption reduces which is the accordance with the results obtained in this study. Furthermore, the investigations showed that the presence of multivalent cations reduces the adsorption of cationic SAILs on negatively charged rock surfaces. Besides, the multivalent cations like, (Ca^2+^) show a stronger tendency to compete for negative adsorption sites as compared to monovalent ions like (Na^+^) and sometimes may also reverse the sign of the surface charge^94^.

In total, the researchers have reported that there are several effective parameters during the adsorption processes namely surfactant nature, solid/fluid system interaction, and the electrostatic attraction between rock surface and surfactant head groups^95,96^. For example, Somoza et al.^97^ investigated the adsorption of 4000 ppm [C_10_mim][TfO], 1 wt% NaOH, and 2 wt% NaCl on the Berea sandstone. They observed that there was no trace of IL in the effluent after 4 PVs of chemical formulation injection (4000 ppm IL, 1 wt% NaOH, 2 wt% NaCl) due to the high adsorption of the IL on the rock surface. They related this observed trend to the fact that the adsorption of ionic surfactants is strongly affected by the rock composition which can convert the rock surface to a negative or positive surface^28^. Besides, they performed another core flooding experiment using a carbonate core plug to investigate the possible adsorption of the IL on the carbonate rock. Their results revealed that the trace of IL was observed in the effluent just after 0.5 PV injection means the lower adsorption of the IL on the carbonate rock due to similar surface charge of both carbonate core plugs and surfactant molecules and possible interaction occurs between the calcite and other minerals containing Mg^2+^ and surfactant molecules^98^.

The results reported by Somoza et al.^97^ and the obtained results in the current investigation are in good agreement with the results reported by Nandwani et al.^99^ that related the observed trend to the strong repulsion force that exists between rock surface and surfactant molecules (both have positive charges) reduces the adsorption, especially for the imidazolium-based ILs.

Besides the facts reported by Nandwani et al.^72^, Ahmadall et al.^57^, Johansen et al.^100^ reported that the rock surface charge plays an important role in adsorption in the light of pH and ionic strength. In detail, this is the electrostatic attraction between the solid surface and the IL head group that controls the IL adsorption and IL molecules migration from the bulk solution to the interface (rock surface)^100^.

The other point besides the amount of adsorption and its mechanism is the shape of the adsorption curve. In detail, the investigations revealed that one of the most common shapes for the adsorption isotherms is S-shape. For example, Austad et al. reported that the ethoxylated surfactants (anionic surfactants with a negatively charged surface) on kaolinite (positively charged adsorbents) displayed an S-shaped isotherm^92^ while Sexsmith et al. reported a similar isotherm pattern (S-shape) for the cationic surfactant of CTAB on cellulosic fiber (negatively charged)^93^. They revealed that 1-hexadecyl-3-methylimidazolium bromide ([C_16_mim][Br]) showed the lowest adsorption on the carbonate surface while the adsorption of the CTAB was greater probably due to the presence of a heterocyclic ring in the SAILs (head group section) leading to stronger repulsion and lower adsorption of the ILs^26^. They reported that the adsorption patterns of examined surfactants are as follow: CTAB > N-hexadecyl-N-methyl pyrrolidinium bromide ([C_16_MPr][Br]) > 1-hexadecyl 3-methyl pyridinium bromide ([C_16_Py][Br] > [C_16_mim][Br].

The last point that must be mentioned is that the difference between the cationic head group of ILs has a significant effect on the adsorption behavior of these surfactants. In detail, imidazolium-based SAIL such as [C_16_mim][Br] showed the minimum adsorption compared with both pyridinium and pyrrolidinium-based SAILs. The reason for this observed trend is that the pyridinium and imidazolium possess an aromatic character which is sp2 hybridized. However, pyrrolidinium is sp3 hybridized and is a non-aromatic cationic head group. Also, pyrrolidinum ILs have a higher dissociation constant than imidazolium and pyridinium-based SAILs which means the higher attraction for pyrrolidinium SAILs under soluble conditions is possible to gain leading to electrostatic attraction towards the negatively charged adsorbents. Besides, the aromatic ring in pyridinium ILs is π-deficient while the aromatic ring in imidazolium-based IL is both π-excessive and π-deficient (presence of two nitrogen atoms)^101^. So, So, as the surfactant contains π-deficient aromatic nuclei and the solid adsorbent has strongly negative sites, the attraction between electron-deficient aromatic nuclei of the adsorbate and negative sites on the adsorbent results in adsorption^102^. As a consequence of this fact, higher adsorption for pyridinium-based SAILs is unpreventable compared with imidazolium-based SAIL.

## Conclusions

The current study is concentrated on the effects of different salts from chloride and sulfate families under the low salinity condition (5000 ppm) except CaSO_4_ which is held constant at 2000 in the presence of crude oils (acidic, neutral, and basic types. Besides, the impact of a new class of surfactant from the ionic liquid (IL) imidazolium family namely 1-dodecyl 3-methyl imidazolium chloride [C_12_mim][Cl] and pH as the operating parameter in the range of 3.5–11 was examined on the interfacial tension (IFT) reduction and IL adsorption on the rock surface. To sum up, the obtained results can be categorized as below:

- Presence of salts as a reducing effect on the IFT reduction especially MgCl_2_ and CaCl_2_.
- Increasing the pH to a value of 11 has a profound effect on the IFT reduction for acidic crude oil (IFT value of 3.1 mN/m) in the presence of Na_2_SO_4_.
- The obtained results revealed that the presence of Na_2_SO_4_ concomitantly with pH variation led to the minimum IFT value for most of the examined cases.
- For all the examined salts except MgCl_2_ and CaCl_2_, the effect of pH enhancement on the IFT reduction due to the saponification process and in-situ surfactant formation is higher than the effect of salts although this trend is reversed for the solutions prepared using MgCl_2_ and CaCl_2_.
- The measured IFT values revealed that besides the in-situ surfactant production, the salting-out effect increased the migration of crude oil active substances toward the aqueous solution and further IFT reduction as the secondary mechanism.
- The presence of IL in the aqueous solution reduced the IFT to the minimum value of 0.08 mN/m for the acidic crude oil/Na_2_SO_4_ solution modified by a pH value of 11.
- The IL adsorption measurements revealed the positive effect of in-situ surfactant preventing the IL adsorption by reducing this value from 0.83 to 0.41 mg IL/g rock using the acidic crude oil. Besides, further measurements revealed the positive effect of a low pH value of 3.5 for IL adsorption reduction if basic crude oil is used. According to these findings, it seems that the pH and crude oil type are two vital parameters that guarantee the success of the EOR method.

## Data Availability

To access the measured experimental data, one can contact Dr. Naser Akhlaghi by contacting email of naserakhlaghi@yahoo.com.
